# Top 100 most cited articles on Patient Reported Experience Measures (PREM): insights and perspectives

**DOI:** 10.1186/s41687-024-00791-z

**Published:** 2024-09-30

**Authors:** Asiya Attar, Kasturi Shukla, Preeti Mulay

**Affiliations:** 1 Symbiosis Institute of Health Sciences, Symbiosis International (Deemed University), Pune, India; 2 Weekend Forever, Pune, India

**Keywords:** Bibliometric analysis, Citations, Patient experience, Patient reported experience measures, PREM

## Abstract

**Purpose:**

Patient experience is fundamental to Patient-Centered Care (PCC). Although prior bibliometric research studies have focused on various aspects of PCC, a comprehensive analysis of PREM articles is required to understand its impact on the clinical practices. This study aims to analyze the top 100 most-cited PREM articles to examine the critical studies and related trends.

**Methods:**

The 100 most cited articles on PREM were gathered from the Web of Science using a combination keyword search approach. The following information was extracted: study design, sample size, topic, number of citations, authorship, country, year of publication, journal title, and dimensions included in these PREM instruments. The VOSviewer software was used to generate graphical bibliometric networks.

**Results:**

The citation count of the top 100 PREM articles varied from 20 to 775 citations. 21 articles had received a minimum of 100 citations. All the articles were in English, and out of these 45% were from the USA. The cross-sectional study (69%) was the most common study design, and the impact of treatment (44%) was the most frequent topic. The common PREM instruments used were customized PREM questionnaires (16%) and HCAHPS (10%).

**Conclusion:**

This bibliometric research showed that the area of PREM is far from being saturated. The authors have attempted to provide an overview of global PREM research. Future research should focus on studies from underdeveloped and developing countries to develop condition–specific PREM tools. Longitudinal researches among special populations and studies in day-care and outpatient settings are recommended in future.

## Introduction

Bibliometric analysis employs a range of techniques to assess the research output, including statistical and quantitative analysis of publications and their citation counts. Researchers can use bibliometrics to find prominent authors, essential journals, research trends, and countries with the highest publication activity. These kinds of analyses can be applied to assess publications from many perspectives, such as the level of an individual author, a team of researchers, a particular work, a particular journal, a field of study, or an academic establishment [1]. The quantity of citations a paper receives is one such indicator used to evaluate its scientific value [2]. The citation count of a paper can be used to gauge its influence on the advancement of knowledge. Several indices, such as the Impact Factor (IF) and the h-index, are used to evaluate a paper as an indirect measure of quality, productivity, and credibility [2, 3]. The number of citations determines the ultimate scientific value of the paper [2, 4]. The citations link the academic work and create a network of scientific literature [3]. Researchers can identify trends and gaps within any field of study by analyzing these citation networks. Hence, this study has utilized bibliometric analysis to determine the top 100 most cited articles on Patient Reported Experience Measures (PREM).

Patient Experience (PE) is one of the crucial elements of Patient-Centered Care (PCC). PE improves healthcare quality in addition to medical expertise and technological advancements [5–7]. It has been reported to be positively correlated with clinical effectiveness, patient safety, self-rated and objectively measured health outcomes, and adherence to the recommended clinical practice [5]. PE data may originate from focus groups, interviews, or massive quantitative datasets drawn from standardized questionnaires. The latter is known as Patient Reported Experience Measures or PREM which collects extensive and expansive patient reports about their experiences of care [6]. In modern healthcare, patients are increasingly involved in their treatment decisions, and expect a high quality of care, making PREM and Patient-Reported Outcome Measures (PROM) essential to provide tailored care to patient’s needs. PROM data is collected before, during and at the end of the treatment procedure, making it difficult to comprehend PE during the treatment process. As PROM are used only to evaluate the outcomes of treatment, these should be supplemented with PREM to enable improvements in care experience. Hence, PREM and PROM should be used in conjunction for an enhanced focus on PCC [8].

Thus far, bibliometric analyses have been reported in various studies related to PCC: patient-centered outcome [9], Health-Related Quality of Life (HRQoL) [10], patient satisfaction [11], condition-specific Patient-Reported Outcome Measures (PROM) [12], Patient-Reported Outcomes (PROs) [13]. Bibliometric analyses were also found on the current trends of patient engagement [14] and the inclusion of patients in the decision-making process of their care [15]. Unlike PROM, PREM is a developing area that is slowly gaining attention. However, with a higher focus on quality of experience during care delivery, there is a huge scope to utilize PREM tools in care pathways. There is a need for studies that report the utility and integration of PREM in regular practice to create a more comprehensive body of knowledge that pertains to the clinical and scientific communities’ interests. Bibliometric studies in this area can bring out the various aspects of the research activities in PREM by highlighting the publications that have shaped the concepts, directed PREM policies, and contributed to the enhancement of the outcomes of clinical practice. Healthcare providers with a high emphasis on PCC can select similar scientific evidence and drive their decisions based on the research results from the authors that PREM researchers have cited most frequently. Owing to the rising interest in PREM, the present study aims to conduct a bibliometric analysis of the top 100 most cited PREM articles, with an emphasis on consolidating and comprehending the global knowledge so far created in the field. The focus of this analysis is to study the characteristics of the top 100 studies and understand the assessment process of PREM. The present study also highlights the most influential articles, and analyzes the research trends and critical themes in PREM literature. Furthermore, the authors have attempted to provide insights into the development and application of PREM instruments in different settings and populations.

## Materials and methods

The Web of Science (WoS) “All Databases” (AD) and “Core Collection” (CC) were used for the article search. (“Patient Reported Experience Measures” OR “PREM”) AND (“patient experience measures”) AND (“patient-reported experience”) AND (“patient experience”) AND (“patient perception”) were used as the search terms. No language restrictions were applied to search articles published until December 2023. Articles that primarily addressed any aspect of PREM were included. Book chapters and conference articles were excluded. Moreover, articles on PROM, patient satisfaction, and any articles that did not cover PREM at all were also excluded as these were beyond the scope of the present study.

Article selection was done until the 100th most cited article was found. The list of 100 most cited PREM articles was displayed in descending order based on the number of citations in WoS-AD. In the case of a draw, the highest citation density (yearly citation count) was used to determine the ranking, with the highest citation count in the WoS-CC [16].

The following bibliometric parameters were extracted from the articles: name of the paper, WoS-AD number of citations, WoS-CC number of citations, WoS-CC citation density (mean number of citations received per year) [16], study objective, study design, sample size, sample age range, number of citations, authorship, country, and continent (depending on the affiliation of the corresponding author), year of publication, journal title, and the PREM instrument or the dimensions of PE used in the scale. Data was double-checked to avoid errors.

Study designs were divided into cross-sectional, systematic review, scoping review, methodological review, narrative review, and scale validation studies. The topic of the studies was categorized into Instrument Development, Cross-cultural validation, Theoretical modeling, Impact of Medical Conditions, and Impact of Treatment.

Bibliometric networks were created using the VOSviewer software. In a co-authorship map, the number of jointly authored publications were used to link the authors to one another once their names were entered into the program as the unit of analysis. Only those authors with two or more publications in the top 100 list were included in this map. A cluster in the network is made up of several nodes that are connected to one another, and each cluster has a distinct color [16]. Key terms are shown using larger circles. Terms with strong partnerships are positioned closer together. Additionally, relationships between items are indicated by lines, with thicker lines denoting a stronger bond between the two items.

## Results

### Search results

The search strategy yielded a substantial dataset of 3239 articles from WoS-AD. These were ranked in descending order of their citation count. After reviewing the 1076 abstracts, 247 full-text studies were selected. 141 studies did not fulfil the inclusion criteria, and 6 full texts were not available. Eventually, a list of the top 100 most cited PREM articles was obtained, and a detailed citation analysis was conducted, which is presented further.

### Citation analysis

The 100 most cited PREM articles received a total of 8945 citations (median: 43, minimum citation: 20, maximum citation: 775) in WoS-AD. A minimum of 100 citations were received by 21 articles. The most cited paper was “Patients’ perception of hospital care in the United States.” authored by Ashish K. Jha, John Orav, Jie Zheng, and Arnold M. Epstein and published in The New England Journal of Medicine (NEJM) in 2008 [17]. The oldest article in our study was authored by Gardner et al. in 1993 [18], and the most recent article was by Naseer et al. in 2021 [19]. Most articles were published in the mid to late 2010s. The overall global trend across various countries indicated that the number of studies has gradually increased over time, with a peak in 2015. The global distribution is elaborated further.

### Geographical distribution

**Fig. 1:**
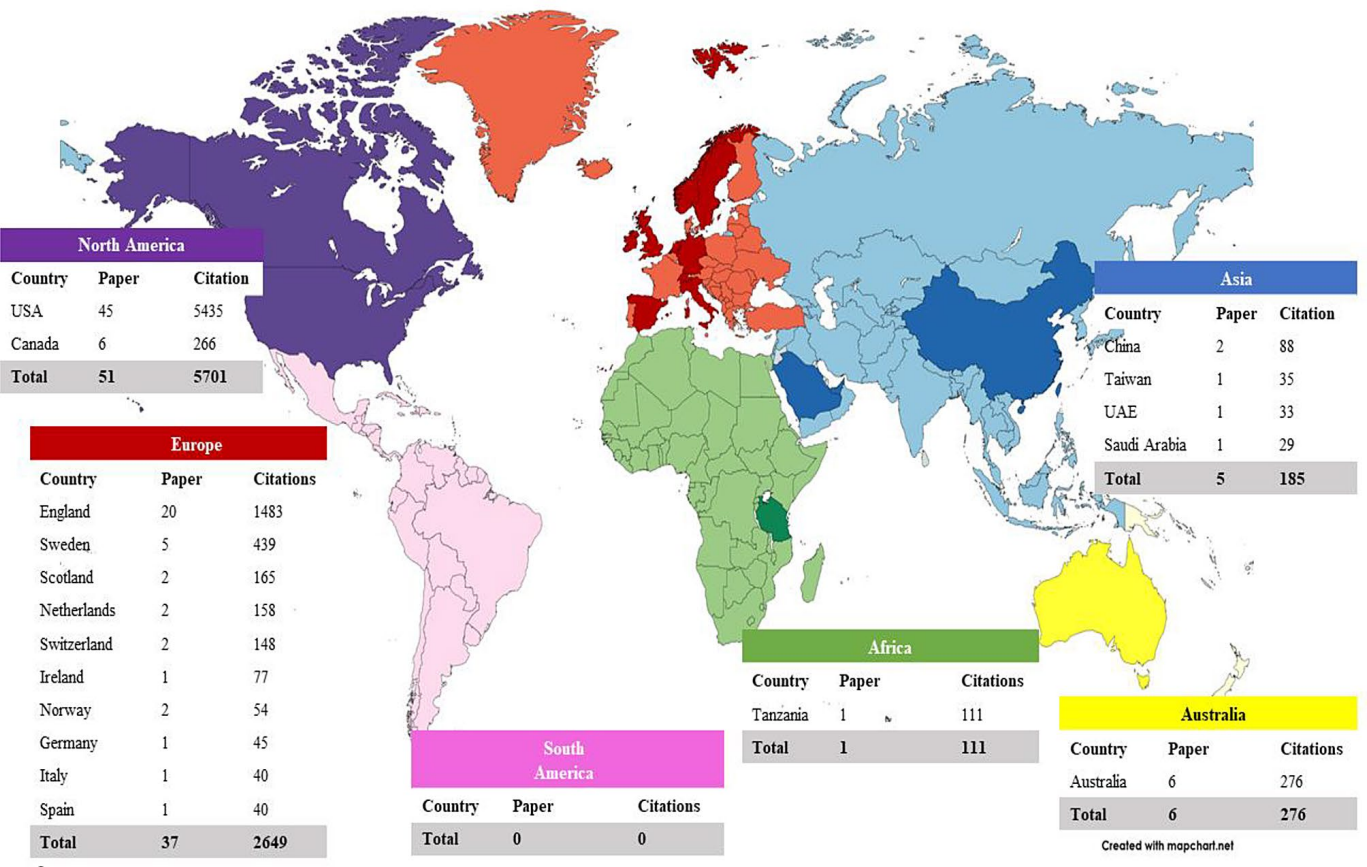
Global distribution of the top 100 most-cited PREM articles. *Source* Authors’ creation; mapchart.net

**Fig. 2:**
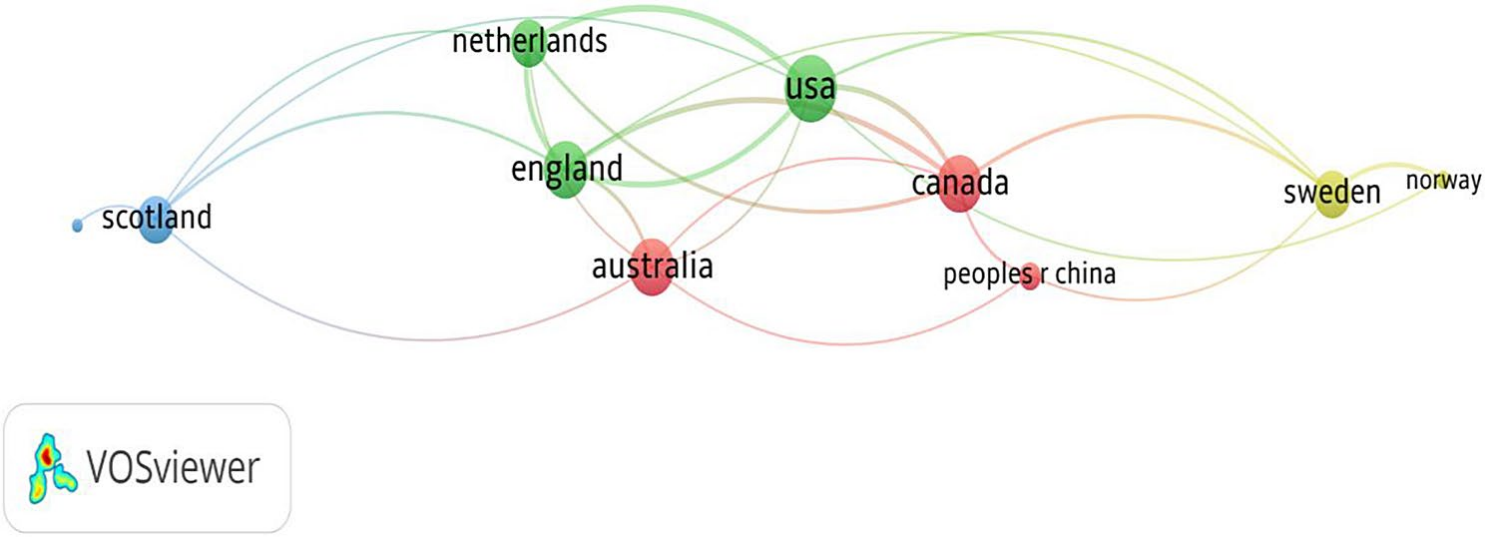
A bibliographic network of the countries in the top 100 most-cited PREM articles. *Source* Authors’ creation; VOSviewer

Figure 1 illustrates the continent-wise distribution of articles and their citations. The highest number of articles was concentrated in two continents, namely North America (51 articles; 5701 citations) and Europe (37 articles; 2649 citations), whereas South America was the only continent without any articles. At the country level, the USA had the highest number of articles and citations (45 articles; 5435 citations). It was observed that European nations (England and Scandinavian countries) were actively working on PREM-related research. We used VoSviewer (Fig. 2) to map detailed networking among the countries.

The links between various nations are depicted in Fig. 2. The map highlights the global research collaborations, with some countries serving as significant hubs for the network. Every nation is represented by a node, and the thickness of the linked lines shows the collaborative nature of each nation. A core hub with strong interconnections formed by the USA, Netherlands, and England demonstrates substantial collaboration. While countries like China, Canada, and Australia are well-integrated into the network, Scotland and Norway appear to be more isolated and have fewer connections. Key authors from these countries are discussed next.

### Authors

**Fig. 3:**
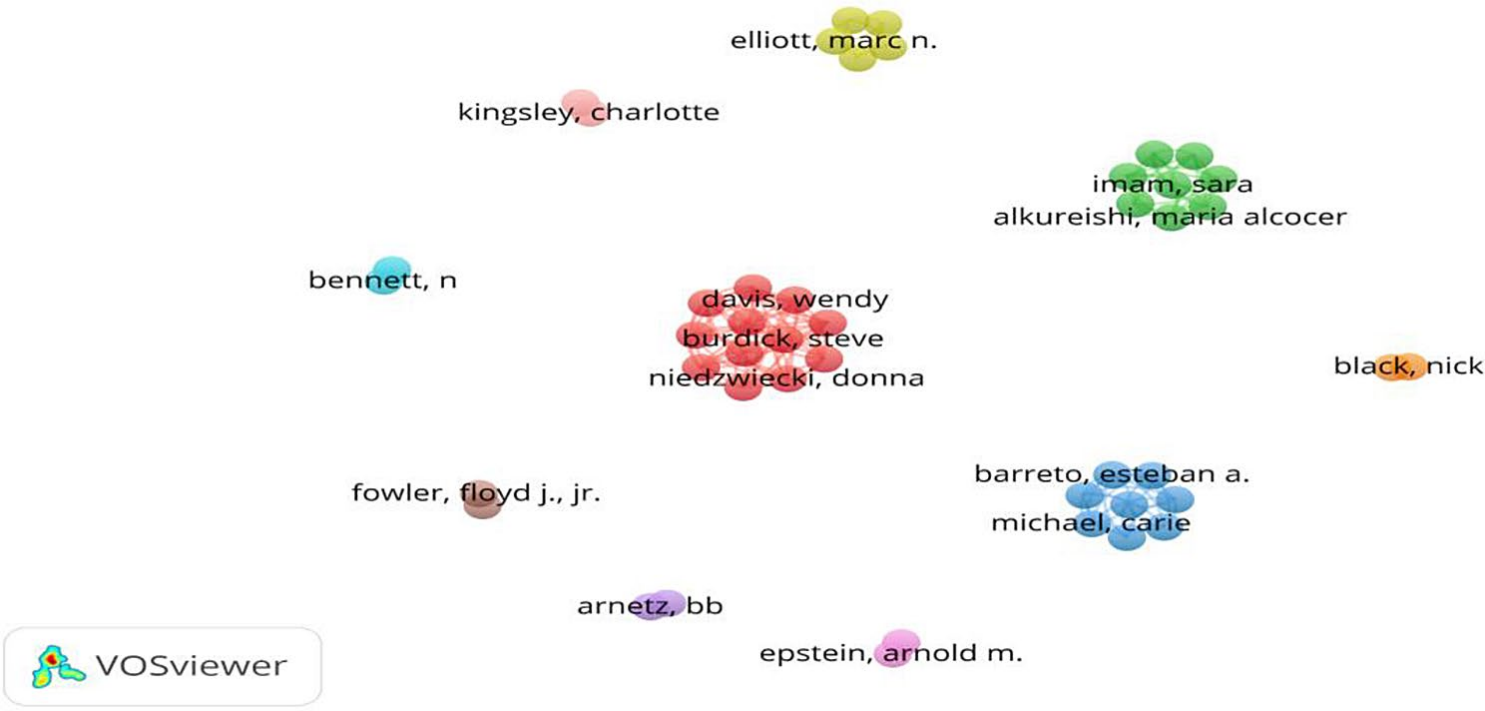
Bibliographic coupling between authors in the top 100 most-cited PREM articles. *Source* Authors’ creation; VOSviewer

An overview of the data on authors who have at least two articles in the top 100 cited PREM research is shown in Table 1. In the top 100 cited PREM articles, these authors have two to four articles with citation counts ranging from 598 to 130. Elliot M.N. is the most prominent author in this field, with four articles and 598 citations. This table shows the evolution of PREM as a result of the highly cited publications of various scholars. Their collaboration links were studied using VoSviewer, illustrated in Fig. 3.

**Table 1:** Authors with at least 2 articles in the top 100 most-cited PREM articles as per WoS-AD

Authors	Number of articles in the list of top 100 PREM	Number of citations in the list of top 100 PREM
Elliot M.N [20–23]	4	598
Giordano, L.A [20, 21]	2	468
Arnetz JE [24, 25]	2	357
Floyd J. Fowler Jr [26, 22]	2	240
Toomey, S.L. [22, 23]	2	130
Schuster, M.A. [22, 23]	2	130
Alan M. Zaslavsky [22, 23]	2	130
David J. Klein [22, 23]	2	130

*Source* Authors’ creation; Web of Science

Figure 3 illustrates a network visualization of author clusters based on collaborative links, such as co-authorship or co-citation. It highlights significant authors and potential collaboration opportunities, with each color representing a closely related group. Strong internal collaboration is noted within these groups, like the prominent yellow cluster featuring Elliott M.N. Larger or central clusters indicate influential researchers or highly interconnected networks, while isolated or peripheral clusters suggest niche study areas or limited collaboration. Some of these impactful studies are discussed next.

### Influential studies

Elliot M.N. has a strong presence that speaks to a substantial body of work and pervasive impact within the domain of PREM research. In one of Elliot M.N.’s renowned publications, “Accelerating Improvement and Narrowing Gaps: trends in Patients’ Experiences with Hospital Care Reflected in HCAHPS public reporting,” the HCAHPS (Hospital Consumer Assessment of Healthcare Providers and Systems) measure was used [20]. The objective of this study was to measure the extent of HCAHPS progress in hospitals that participated in the public reporting for the second and the fifth year. A limitation of the study is that since it leaves out important facets of patient care, the standardized format of the HCAHPS survey may restrict its depth of coverage. Another factor that could have created a response bias was self-reported data.

A study entitled “Development, Implementation, and Public Reporting of the HCAHPS Survey” by L.A. Giordano used the data from the HCAHPS survey to investigate how healthcare communication affects PE [21]. The dataset of this study included responses from a number of US hospitals between the years 2012 and 2016. This study has highlighted the particular circumstances in which healthcare professionals could improve their communication while enhancing patient satisfaction. However, its cross-sectional design made it difficult for the researchers to prove a long-term association between communication styles and patient outcomes. There was also a possibility of selection bias and participation bias since all the hospitals were not a part of this research study. Therefore, longitudinal studies with a greater number of participating hospitals are recommended to gain a deeper understanding of the connections between such variables.

Research by Arnetz, J.E. frequently examined the connection between PE and improvements observed in the hospital setting. One such prominent study is “The Development and Application of a Patient Satisfaction Measurement System for Hospital-Wide Quality Improvement [24].” The research aimed to design a valid and reliable technique for measuring patient satisfaction, identify factors that influence patients’ perceptions of quality and test patient satisfaction. This was done to analyze changes that occur after employing a quality improvement program. The research was noteworthy for illustrating the influence of environmental elements on PE using the Quality Work Competence (QWC) patient satisfaction measure. Despite its contributions, the study had drawbacks. It relied on a single survey type and did not account for potential confounding variables such as hospital policy and staffing levels. Moreover, the research design precluded an analysis of the long-term effects of the quality improvement projects. There has been a significant improvement in the comprehension of PREM in healthcare due to these articles. It was, therefore, necessary to analyze the journals that disseminated PREM articles to comprehend their impact and reach. This is elaborated in the next subsection.

### Publications

*BMC Health Services Research* had highest published articles (5) with 181 citations. However, the most frequent publication was done by *Medical Care* (4 articles) and *Pediatrics* (4 articles). An analysis of the study designs was necessary to comprehend how these publications were disseminated and reached a larger audience of researchers. This is discussed in the next subsection.

### Study designs

The top 100 most cited articles included 82 primary studies. Out of these, 13 studies have reported newly validated PREM scales. The study designs and the number of articles that used these designs are listed in Table 2. Sample sizes range of the primary study design were as follows: Cross-Sectional (Maximum: 4,822,960; Minimum: 11), Validation (Maximum: 49,812; Minimum: 13).

**Table 2:** Summary of study designs used in the top 100 most-cited PREM articles

Study design	Number of articles	Number of citations	Citation ratio^a^
Cross-sectional	69	5170	74.9
Systematic review	12	1472	122.6
Validation	13	1372	105.5
Scoping review	1	33	33
Methodological review	1	428	428
Narrative review	4	450	112.5
Total	100	8925	876.5

^a^Number of citations/numbers of articles

*Source* Authors’ creation

The cross-sectional study (69 articles; 5170 citations; 74.9 citation ratio) was the most-cited study design. This massive volume indicates that, in order to look at data at a specific point in time and create an overview of PE, cross-sectional studies are the commonly employed in PREM research. Nevertheless, diversifying the study designs with a higher number of longitudinal research shall supplement rigor and generalizability.

The validation studies have a citation ratio of 105.5 with 13 publications and 1372 citations. The validity and reliability of these studies are primarily responsible for the widespread adoption of PREM instruments and the trust of experts in these assessments. The high citation ratio serves as evidence of their important contribution in creating and validating credible PREM scales.

There are only 12 systematic review articles, but these have high citation ratio (122.6). This implies a high research influence of the systematic review articles. Additionally, it offers a comprehensive and empirically validated insights from the body of recent research.

Scoping and narrative reviews are less common, but they have varying degrees of impact. The single scoping review has a lower citation ratio (33), which may indicate a narrowly focused or recently developed topic of the study.

The citation ratio of a narrative review with four articles and 450 citations is 112.5. However, among all the articles with only one article, methodological reviews have the highest ratio (428), indicating the significance of methodological advancements and insights in directing future research paths. The fact that the various types of review articles receive a lot of citations suggests that they are crucial for integrating different viewpoints and offering context. Further, the characteristics of the reviewed articles were analyzed by categorizing them under 5 study topics. This is explored further in the next subsection.

### Study topics

Table 3 illustrates the classification of the study topics. The most cited topic was the impact of treatment (articles: 44; citations: 4499; citation ratio: 102.2). Articles on cross-cultural validation had the highest citation ratio (218.7). 70 articles investigated the impact of medical conditions and treatment. Cancer (8%) was the most common condition investigated, followed by mental illness (7%), maternity care (2%), diabetes (2%), and hypertension (2%). Investigation of oral conditions, respiratory conditions, obesity, and neurologic conditions are less common.

**Table 3:** Summary of the study topics of the top 100 most-cited PREM articles

Topic	Number of articles	Number of citations	Citation ratio^a^
Instrument development	25	1945	77.8
Cross-cultural validation	4	875	218.7
Theoretical modeling	1	43	43
Impact of medical conditions	26	1519	58.4
Impact of treatment	44	4499	102.2
Total	100	8881	500.1

^a^Number of citations/numbers of articles

*Source* Authors’ creation

Cross-cultural validation has the largest citation ratio among the four publications, with 875 citations. The relatively higher ratio emphasizes the significance of assessing PREM and adapting them to diverse socio-cultural settings. This was done to ensure the applicability and appropriateness for a spectrum of populations.

The impact of treatment is the most investigated topic, with 44 articles and 4499 citations (citation ratio: 102.2). This high citation count and ratio demonstrates the importance of evaluating the association between treatment plans, PE and overall quality of care delivery.

The impact of medical conditions is another significant topic, with 26 publications yielding 1519 citations; the citation ratio is 58.4. This area of study examines how various medical issues affect PE. It also provides details regarding the particular challenges faced by the various patient populations like differently abled, geriatric and female patients.

Theoretical modeling has a citation ratio of 43, with only one article having forty-three citations. The specialized nature of theoretical work frequently forms the basis for more extensive empirical investigations. Yet, a relatively lower influence was observed since this article could not obtain many citations.

These 5 categories make it easier to comprehend the context in which PREM instruments have been used. However, an understanding of the types of PREM instruments used in these studies was also necessary. It is explored in the next subsection.

### Commonly used PREM instruments

Table 4 summarizes the application of the various PREM scales and the dimensions drawn from other PREM instruments (such as CAHPS or Picker) (articles: 16, citations: 1471; citation ratio: 91.9).

**Table 4:** Summary of the commonly used PREM instruments in the top 100 most-cited PREM articles

Commonly used PREM instruments	Number of articles	Number of citations	Citation ratio^a^
Consumer Assessment of Healthcare Providers and Systems (CAHPS)	8	968	121
Hospital Consumer Assessment of Healthcare Providers and Systems (HCAHPS)	10	782	78.2
Picker Patient Experience Questionnaire	4	698	174.5
PREM instrument not specified, but PE dimensions used	16	1471	91.9
Primary Care Assessment Tool	4	160	40
Survey of Healthcare Experiences of Patients (SHEP)	2	103	51.5

^a^Number of citations/number of articles

*Source* Authors’ creation

The most common PREM instrument was the Hospital Consumer Assessment of Healthcare Providers and Systems (HCAHPS) (articles: 10, citations: 782; citation ratio: 78.2) and the second most commonly applied scale Consumer Assessment of Healthcare Providers and Systems (CAHPS). CAHPS had multiple modified and validated versions available, namely: CAHPS, SHEP-CAHPS, and child CAHPS (articles: 8, citations: 968; citation ratio: 121).

The complexity of PE research is generally highlighted by the variations in the citation impact of the different PREM instruments. The Picker Patient Experience Questionnaire (PPEQ) and the CAHPS are the two PREM tools with wide applicability and a reliable and standardized methodology. This information can assist researchers in selecting suitable PREM instruments with relevant dimensions to develop newer tools.

## Discussion

The current study covering the top 100 most-cited PREM articles is based on research articles, and reviews through meta-analysis, which yielded data on research trends, regional distribution, and other relevant bibliometric variables. Since PREM are large, multifaceted constructs, this study identified the highest possible number of articles in the field using keyword combinations. The selection of WoS-AD made it possible to compile a sizable number of citations from academic journal publications [27, 28]. The characteristics of each article were analyzed to highlight the geographic distribution, citation analysis, authors and their influential studies, study trends and designs, and commonly used PREM instruments.

### Regional Disparity

Similar to the previous PCC research [9–15], majority of the publications were from North America (USA and Canada) and Europe (mostly England and Sweden) (Fig. 1). These nations host the top global research centers and allocate significant financial resources [29, 30]. These nations have developed significant PREM instruments, which explains their high citations. The negligible number of articles from developing and underdeveloped countries in the top 100 list suggests that there is either a paucity of research on PREM or an under-appreciation of utilizing PREM in healthcare. Further, all the articles were in English, implying a paucity of research evidence in other languages. This creates an opportunity for researchers in other settings who can create and validate the translated versions of these tools.

### Citations

The highest citation in the top 100 list was 775, received by an article authored by Ashish Jha [17], and the lowest citation was 20. It is observed that the number of citations is influenced by the year of publication. The citation rates of older articles tend to be higher than those of more recent ones. Recent research articles require a larger time frame to gather a higher number of citations which can be tracked by future studies. The process of publishing a consistent, highly cited work is difficult and entails originality, excellence, significance, usefulness, and timely publication [31]. Numerous underlying factors also affect these citation counts, such as the authors’ networks, the journals in which they publish, and their institutional relationships. Self-citations were included in the citation count in this study. Despite the possibility of being misused to boost bibliometric indicators, self-citations have no discernible impact on bibliometric studies [32–34]. The IF is allegedly unaffected by, nor does it correlate with, the self-citation metrics reported in Journal Citation Reports (JCR) [35].

### Author diversity

The most active and often quoted authors are from the United States, England, Scandinavian countries, Australia, and Canada (Fig. 2). Authors from several developed countries have fewer articles but with a relatively high citation count. The author with the maximum number of articles and highest citations was Elliot MN (Table 1). With a total of 4 publications and 598 citations, this author impacted scholars globally and added to the understanding of PREM. Multiple authors have advanced the discipline by developing new PREM instruments, publishing cross-cultural validations, and developing suitable tools for various social contexts. Through strong networks, authors create a worldwide impact, as evident by the VOSviewer map (Fig. 3). Hence, to enhance the impact, research must be conducted collaboratively at a high scale and disseminated strategically.

### Publication gap

Studies on PE, a key indicator of PCC, began in 1995 [36]. The oldest article in our review is from 1993, but the most recent is from 2021. There is a span of just 28 years between their publication, which is a relatively short period compared to other bibliometric studies on PCC, which often cover longer time spans [16]. This suggests that the field is still developing, with a potential for growth and exploration in the future, which should be acknowledged and acted upon by the publishing journals.

Even though *BMC Health Services Research* has published 5% of the top 100 most cited PREM articles, more journals supporting PREM research are required. There is a need to increase the global adoption of PREM tools, foster diverse perspectives, and identify and improve important characteristics of PREM research.

### Study design

The sample size is characteristic that best describes the history of the most relevant investigations on the subject, and it also has ethical implications for the approval and execution of research projects [34]. There are only a few pediatric studies, whereas PREM research on the elderly population is gaining more popularity. Hence, a clear disparity remains in the representation of particular cohorts, such as the differently-abled, geriatrics, and female population. Cross-sectional epidemiological studies had the largest sample sizes leading to a high generalizability of their results. The cross-sectional study design was the most commonly employed (Table 2). The importance of developing PREM tools is highlighted by the substantial number of validation studies. One area in PREM research that needs improvement is the lack of cohort and longitudinal studies in the top 100 list. These studies generate a high degree of evidence (such as randomized control trials and cohort studies) and hence require a higher emphasis in future research.

### Topic of articles

Most articles focused on the impact of medical conditions and their treatment on the PREM among in-patients who are admitted for more than 24 h (Table 3). There were correlations between a higher prevalence of medical disorders and poorer Patient Reported Experience (PRE). It should be noted that depending on the treated medical condition and the individual perspective, medical treatment has varying effects on the PRE. Medical treatment was found to improve PREM for diabetes, cancer, mental illness, and maternity care. Research on the impact of diseases and treatment on PRE facilitates the identification of disparities, helps to allocate resources, assists decision-making, and supports the assessment of programs, policies, and services [6, 37]. However, in our study, there was a dearth of PREM-related articles amongst patients suffering from medical conditions, such as chronic illnesses, dental problems, and conditions that are largely dealt with in an outpatient or a day-care setting. This gap underscores the need for potential studies particularly to explore and analyze PRE in the patients under intensive care, or availing services in outpatient care and day-care procedures.

### Instruments

Healthcare professionals have used some aspects of PE and dimensions from different PREM instruments or modified the existing PREM instrument to develop a self-administered questionnaire for the specific condition. The most commonly used PREM instruments were the PPEQ, CAHPS, and HCAHPS (Table 4). In addition to being the first tools created, they have also demonstrated their validity, dependability, and responsiveness in a variety of research settings. The shorter versions of these scales have been validated to reduce the application time and, increase their applicability. These tools also allow for investigations across a variety of ages. The studies lacked comparable and standardized age groups, which prevented additional age-range analysis. Studies varied in how they presented age data: some used specific age ranges (e.g., 7 years old), some used broad ranges (e.g., 18–65 years old), whereas some only provided the mean age with standard deviation. It was also observed that PREM instruments intended for specific illnesses were frequently utilized to record patient experiences related to different diseases. For instance, instruments developed and validated for chronic medical conditions were utilized to assess PE in dentistry due to the unavailability of relevant scales. Therefore, it is essential to develop and validate PREM across various clinical specialties for an appropriate assessment of PRE and PE.

## Conclusion

This bibliometric analysis reemphasizes the need for continued research on PREM and its integration with patient care. The study also highlights the role of PREM, provides the trends, hotspots, and a broad overview of the global PREM-related research.

Firstly, it was observed that PREM research is largely concentrated in the USA, Netherlands, and England but has not spread across the developing and the under-developed nations. This also explains the predominance of the English language in PREM scales. Secondly, in the author’s networking analysis of the top cited papers, Elliott M.N. from the USA emerged to have the most cited studies through collaborative research. Thirdly, the most common research design in these studies was cross-sectional, with ‘the impact of treatment’ being the commonest topic. Fourthly, the commonly used instruments were the customized PREM tools and the HCAHPS scale. Additionally, the availability and usability of PREM instruments in diseases/conditions and specific populations were the main subjects of the research hotspots, which shows a low focus on the tools that can be utilized for generic populations.

The knowledge gaps can be plugged if future research focuses on developing and validating PREM tools for various health conditions and specialities, such as chronic illnesses and dental diseases, with wider and more relevant dimensions coverage. The journals focusing on PCC should also include and highlight PREM in their scope to encourage research in this area. The literature on PE and PREM from developing and underdeveloped nations should focus on validating PREM tools in languages beyond English. This will provide a rich inventory to researchers for multi-cultural and cross-cultural studies. Additionally, future studies should focus on outpatient and daycare settings, female patients, geriatric groups, and specially-abled populations using cross-sectional and longitudinal designs.

## Data Availability

All data generated or analyzed during this study are included in this published article.
