# Correction: Top 100 most cited articles on Patient Reported Experience Measures (PREM): insights and perspectives

**DOI:** 10.1186/s41687-024-00801-0

**Published:** 2024-10-22

**Authors:** Asiya Attar, Kasturi Shukla, Preeti Mulay

**Affiliations:** 1 Symbiosis Institute of Health Sciences, Symbiosis International (Deemed University), Pune, India; 2 Weekend Forever, Pune, India

**Correction to: Journal of Patient-Reported Outcomes 8, 114 (2024)**

10.1186/s41687-024-00791-z

Following publication of the original article it was reported that Preeti Mulay was mistakenly assigned affiliation 1 in addition to affiliation 2.

The incorrect affiliations were:

Preeti Mulay^1,2^

^1^Symbiosis Institute of Health Sciences, Symbiosis International (Deemed University), Pune, India

^2^Weekend Forever, Pune, India

The correct affiliations are:

Preeti Mulay^2^

^2^Weekend Forever, Pune, India

The original article has been updated.

